# An Ab Initio Study of Lithization of Two-Dimensional Silicon–Carbon Anode Material for Lithium-Ion Batteries

**DOI:** 10.3390/ma14216649

**Published:** 2021-11-04

**Authors:** Alexander Galashev, Alexey Vorob’ev

**Affiliations:** Institute of High Temperature Electrochemistry, Ural Branch, Russian Academy of Sciences, Sofia Kovalevskaya Str. 22, 620990 Yekaterinburg, Russia; vorobev@ihte.uran.ru

**Keywords:** anode material, energy and electronic properties, graphene, lithium, silicene

## Abstract

This work is devoted to a first-principles study of changes in the structural, energetic, and electronic properties of silicene anodes during their lithium filling. Anodes were presented by silicene on carbon substrate and free-standing silicene. The ratio of the amount of lithium to silicon varied in the range from 0.06 to 1.125 for silicene on bilayer graphene and from 0.06 to 2.375 for free-standing silicene. It is shown that the carbon substrate reduces the stability of the silicene sheet. Silicene begins to degrade when the ratio of lithium to silicon (*N*_Li_/*N*_Si_) exceeds ~0.87, and at *N*_Li_/*N*_Si_ = 0.938, lithium penetrates into the space between the silicene sheet and the carbon substrate. At certain values of the Li/Si ratio in the silicene sheet, five- and seven-membered rings of Si atoms can be formed on the carbon substrate. The presence of two-layer graphene imparts conductive properties to the anode. These properties can periodically disappear during the adsorption of lithium in the absence of a carbon substrate. Free-standing silicene adsorbed by lithium loses its stability at *N*_Li_/*N*_Si_ = 1.375.

## 1. Introduction

Modern automobile manufacturing and large stationary energy storage devices (used to smooth out loads in smart grids) require cheap and high-capacity batteries. There is an urgent need to develop high-performance anodes for lithium-ion batteries (LIBs). Graphite anodes currently used in LIBs have a low theoretical specific capacity (372 mAh·g^−1^), which does not allow the creation of LIBs with a high energy density [1]. Low specific capacity is not the only drawback of a graphite electrode. The observed accelerated loss of lithium reserves and graphite exfoliation are associated with gas evolution at this electrode. LIBs capable of developing high power are required, for example, for road transport. A high theoretical capacity of silicon (4200 mAh·g^−1^) and a suitable operating voltage make it one of the most promising anode materials for a LIB [2]. However, bulk crystalline or amorphous silicon cannot serve as a good anode material for LIB because of the large (up to 300%) volume change during cycling and low electrical conductivity [3]. Increasing the electrical conductivity of the electrode facilitates a fast LIB charging process. A large change in the volume of silicon during the electrode charging/discharging results in the cracking and subsequent disintegration of the anode. Therefore, a LIB quickly becomes ineffective. The promising efficiency of silicene as a LIB material has been shown in several theoretical studies [4,5,6]. It was also shown that the lithium filling of both free-standing and two-layer silicene leads to its mechanical destruction, so the theoretical capacity of silicene cannot be achieved. An ab initio study of the adsorption of lithium on free-standing single-layer silicene showed that the specific capacity of such an anode, which did not experience bond rupture, should be 1196 mAh·g^−1^ [7]. A similar study performed for two-layer free-standing silicene showed that a bond rupture, initiated as a result of lithium adsorption, limits the specific capacity of such electrode at the level of 955 mAh·g^−1^ [8].

At the same time, it is shown that silicene has high adhesion to metal substrates such as Al, Ag, Au, Cu, and Ni [9,10,11,12]. The backing made of high-strength material acts as a framework and can enhance the mechanical strength of silicene. Atomic force microscopy showed that a thin stack of graphene layers (fewer than five) has a Young’s modulus of about 0.5 TPa, whereas a Young’s modulus ~1 TPa corresponds to monolayer graphene [13]. The mechanical properties of graphene are not inferior to those of diamond, for which Young’s modulus is in the range from 0.41 to 0.59 TPa [14,15]. Thus, bilayer graphene can serve as a solid base for silicene when used as the LIB anode material. Although density functional theory (DFT) calculations indicate that the distribution of pentagon–heptagon pair grain boundaries affects the internal strength of graphene [16]. Typically, the grain boundary effect on the mechanical properties of polycrystalline graphene is important at high temperatures. It is assumed that siligraphenes g-SiC_2_ and g-SiC_3_ with specific capacities of 1286 and 2090 mAh·g^−1^, respectively, can be created from two-dimensional Si/C composites [17]. Another important quantity characterizing the strength of the combined silicene–graphene anode is the adhesion energy between the silicene sheet and the graphene substrate. Since this energy, as a rule, is significantly inferior to the energy of adhesion between the silicene sheet and any of the metal substrates, the mechanical strength of the silicene–graphene pair needs careful study.

The purpose of this work is to investigate the possibility of using a silicene–graphene composite material as an anode of a lithium-ion battery, as well as to calculate the important energy and structural and electronic characteristics during the system operation.

## 2. Materials and Methods

**Figure 1:**
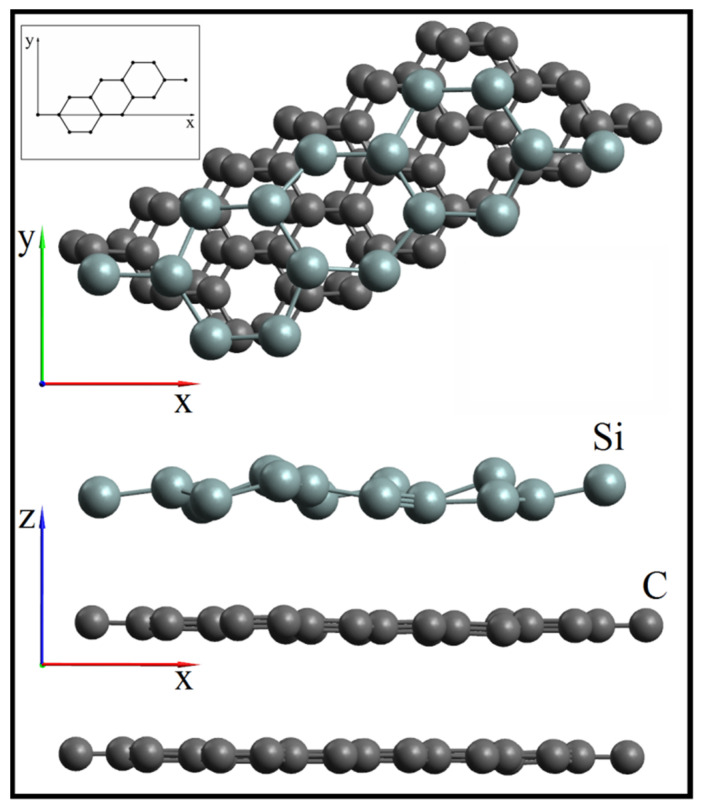
Top and side views of the configuration of the “silicene-bilayer graphene” system after geometric optimization; the top insert shows the direction of the x and y axes relative to the silicene 4 × 2 supercell.

The present DFT study of polyatomic adsorption of lithium was carried out on models of a stand-alone (free-standing) silicene sheet and a silicene sheet located on bilayer graphene. Figure 1 shows the geometric structure of the silicene–bilayer graphene system, and the insert in the top of this Figure reflects the direction of the *x* and *y* axes relative to the silicene 4 × 2 supercell. The silicene sheet for the systems under consideration was modeled on the basis of a 4 × 2 supercell (16 silicon atoms) increased by 4%, with two sublattices (lower and upper) separated from each other by a distance of 0.44 Å. At the same time, the graphene substrate was specified by two 8 × 4 supercells (32 carbon atoms) reduced by 4% and separated by a distance of 3.25 Å in the z-direction. As shown in [10], a 5% scaling of the superlattice of a combined silicene-containing two-dimensional material does not lead to a significant change in its electronic properties. Layers of graphene (formed by carbon hexagons) were stacked in parallel, determining the stacking regularity of AB …, i.e., according to Bernal’s packaging. A system consisting of two graphene layers already reflects quite well the energy properties of a bulk graphite substrate [12,18]. The spatial translation period in the z-direction in all considered cases was 35 Å.

When studying the adsorption of lithium, the ratio of the number of deposited lithium atoms to the number of silicon atoms was from 0.0625 to 1.125 for silicene on a carbon substrate and from 0.0625 to 2.375 for a free-standing silicene sheet. The location of lithium atoms in the system was set as follows: (i) the first 3 (6 for a free-standing silicene sheet) atoms were deposited in a position above the hexagonal ring; and (ii) further deposition took place over silicon atoms. Moreover, the deposition of lithium was carried out on both sides of the free-standing silicene sheet. In the case of silicene on a graphene substrate, the deposition occurred only over the silicene sheet (on one side).

The generalized gradient approximation in Perdew–Burke–Ernzerhof form was used to represent the exchange–correlation functional [19]. A geometric optimization using an approximation in the form of DRSLL [20], taking into account the Van der Waals interaction, was carried out for all considered systems. The dynamic relaxation of atoms continued until the change in the total energy of the system became less than 0.001 eV. The cutoff energy of the plane wave basis set was 400 Ry. The Brillouin zone was specified by the Monkhorst–Pack method [21] using 10 × 10 × 1 k-points. After geometric optimization, the resulting systems were tested for temperature stability using ab initio molecular dynamics. The calculations were carried out using a Nosé–Hoover thermostat, due to which the temperature was maintained at 293 K [22]. The time step length was 1 fs, and the calculation duration was determined by 1000 time steps. The resulting systems were subjected to geometric optimization. Band structures were calculated to determine the electronic properties of materials. These calculations were performed using the Siesta software package [23]. Pseudopotentials and basis sets for calculations were taken from [24].

For the considered systems, the following characteristics were calculated:

1. The adhesion energy between the lithium coating and the rest of the system was determined according to the expression:
  (1) $$E_{a}^{\mathrm{Li}}=-\frac{E_{\mathrm{tot}}-E_{\mathrm{Li}}-E_{1}}{N_{\mathrm{Li}}}$$
  where *E*_tot_ is the total energy of the entire system, *E*_Li_ is the energy of separately taken entire lithium subsystem, *E*_1_ can represent both the energy of free-standing silicene and that of the “silicene on a carbon substrate” system, and *N*_Li_ is the number of lithium atoms in the system.

2. The formula for the bond energies between all the atoms of the system, the atoms in the silicene sheet, and the lithium coating were determined according to the expression:
  (2) $$E_{b}^{\mathrm{Tot}/\mathrm{Si}/\mathrm{Li}}=-\frac{E_{0}-N_{\mathrm{Li}}E_{1\mathrm{Li}}-N_{\mathrm{Si}}E_{1\mathrm{Si}}-N_{C}E_{1C}}{N_{\mathrm{Li}}+N_{\mathrm{Si}}+N_{C}}$$
  where *E*_0_, depending on the calculated energy, is the total energy of the entire system, the energy calculated for a silicene sheet, or the energy of the lithium subsystem; *E*_1Li_, *E*_1Si_, and *E*_1C_ are the energies calculated for single lithium, silicon, and carbon atoms, respectively; and *N*_Si_ and *N*_C_ are the numbers of silicon and carbon atoms in the system, respectively. The calculation of the total bond energy was carried out for the entire system, while calculations of bond energy for a silicene sheet and that for the lithium subsystem were carried out at zero values of (*N*_Li_, *N*_C_) and (*N*_Si_, *N*_C_), respectively.

3. Open-circuit voltage:
  (3) $$\mathrm{OCV}=\frac{E_{\mathrm{Si}}+E_{1\mathrm{Li}}N_{\mathrm{Li}}-E_{\mathrm{tot}}}{n_{e}}$$
  where *E*_Si_ is the energy calculated for a silicene sheet and *n_e_* is the number of valence electrons in the system.

## 3. Results and Discussion

Table 1 and Table 2 show the system characteristics calculated by the method of ab initio molecular dynamics at the temperature of 293 K depending on the ratio of lithium to silicon. Among the characteristics of silicene on a carbon substrate, calculated according to the lithium adsorption, we determined: (1) the total bond energy of all atoms in the system (*E*_b_); (2) the bond energy of silicon atoms in the silicene sheet (*E*^Si^_b_); (3) the bond energy between Li atoms in the lithium subsystem (*E*^Li^_b_); (4) the average bond lengths between silicon and lithium atoms (Si-Li); (5) the average bond lengths between silicon atoms (Si-Si); (6) the average distances between lithium atoms (Li-Li); and (7) the total Voronoi charge, calculated for lithium atoms (*Q*_v_).

As can be seen from Table 1, the *E*^Li^_a_ value experiences fluctuations and decreases as the *N*_Li_/*N*_Si_ ratio increases. In the following, we will refer to the *N*_Li_/*N*_Si_ ratio as the adsorption index. The difference in the *E*^Li^_a_ values at *N*_Li_/*N*_Si_ = 0.0625 and 1.125 reaches 45%. The Si-Li bond length first increases, as the adsorption index increases, and reaches a maximum at *N*_Li_/*N*_Si_ = 0.1875. Then, however, it rapidly decreases when approaching the value *N*_Li_/*N*_Si_ = 0.3125, and further ocsillations in the Si-Li bond length are observed as the adsorption index continuous to increase. The Si-Si bond length increases nonmonotonically during the adsorption of Li atoms. Its value at *N*_Li_/*N*_Si_ = 1.125 is 5% higher than at *N*_Li_/*N*_Si_ = 0.0625. The behavior of the Li-Li distance with an increase in the number of adsorbed Li atoms is vibrational. The value of this quantity at *N*_Li_/*N*_Si_ = 1.125 turns out to be 3.3% lower than at *N*_Li_/*N*_Si_ = 0.625.

Table 2 shows similar characteristics calculated for a free-standing silicene sheet with the addition of the band gap (BG). For free-standing silicene, the *E*^Li^_a_ value decreases nonmonotonically with an increase in *N*_Li_/*N*_Si_, i.e., it behaves similarly to the system where silicene is located on bilayer graphene. The difference in the values of this characteristic, determined at *N*_Li_/*N*_Si_ = 0.0625 and 2.375, was 72%. The Si-Li (*N*_Li_/*N*_Si_) dependences for these systems are also quite similar. For free-standing silicene, after passing through the main maximum (at *N*_Li_/*N*_Si_ = 0.1875), no strong fluctuations are observed with a further decrease in the values of this function. In this case, the Si-Si bond length continues to increase almost monotonically with an increase in adsorption index. The Si-Si value at *N*_Li_/*N*_Si_ = 2.375 is 6.5% higher than at *N*_Li_/*N*_Si_ = 0.0625. The Li-Li average distance decreases nonmonotonically with an increase in adsorption index values. At the end point of the interval under consideration, the Li-Li distance is 4.2% less than at the point *N*_Li_/*N*_Si_ = 0.625. At the value *N*_Li_/*N*_Si_ = 0.25 and in the range 0.375 ≤ *N*_Li_/*N*_Si_ ≤ 1, the free-standing lithiated silicene becomes a narrow-gap semiconductor, while at other values of adsorption index considered, it exhibits conductive properties.

**Figure 2:**
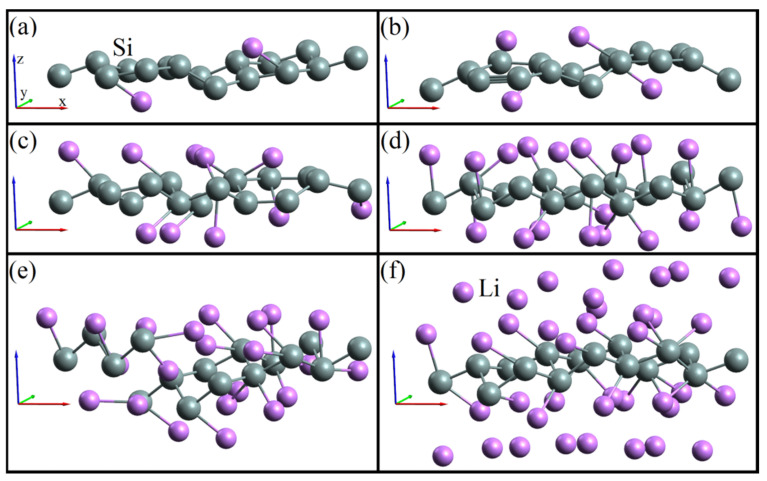
Geometric structures of a single-layer free-standing silicene, which adsorbed lithium in relation to silicon: (**a**) 0.125, (**b**) 0.25, (**c**) 0.625, (**d**) 1.125, (**e**) 1.375, and (**f**) 2.375.

Figure 2 shows the geometric structures of a silicene sheet upon adsorption of lithium with respect to silicon (a) 0.125, (b) 0.25, (c) 0.625, (d) 1.125, (e) 1.375, and (f) 2.375. In an autonomous silicene sheet, upon the adsorption of lithium atoms, geometric rearrangements occur; this is how the lengths of Si-Si bonds change from 2.282 to 2.481 Å. The formation of defects due to the penetration of lithium in the silicene sheet (when Li atoms are together with Si atoms) is observed at the adsorption index of 1.375 (Figure 2), which is higher than that obtained for the silicene channel (1.11) in [8]. Given a simple proportion in which the specific capacity of 4200 mAh·g^−1^ corresponds to the ratio of Li atoms to Si atoms equal to 4.4, we obtain the specific capacity for the anode (1312 mAh·g^−1^) formed by defect-free single-layer free-standing silicene. This value is almost 9% higher than the similar characteristic defined in [7].

**Figure 3:**
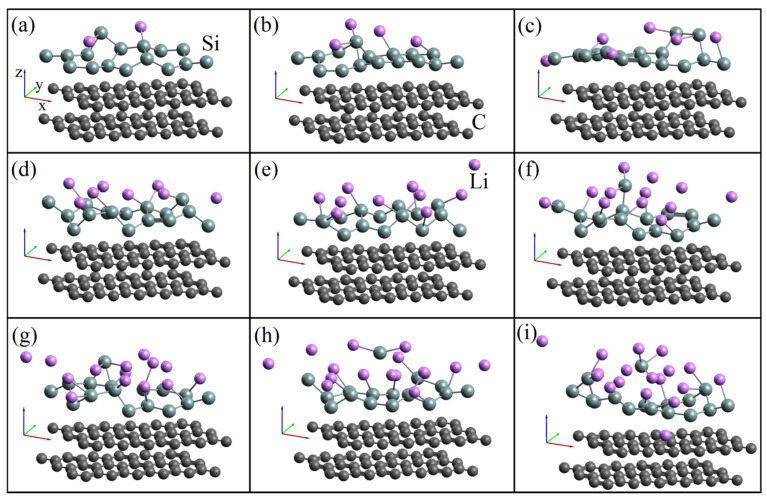
Geometric structures of “silicene-bilayer graphene” systems, which adsorbed lithium in relation to silicon: (**a**) 0.125, (**b**) 0.25, (**c**) 0.375, (**d**) 0.5, (**e**) 0.625, (**f**) 0.75, (**g**) 0.875, (**h**) 1, and (**i**) 1.125.

Figure 3 shows the geometric structures of a silicene sheet on a carbon substrate in cases of lithium adsorption in relation to silicon (a) 0.125, (b) 0.25, (c) 0.375, (d) 0.5, (e) 0.625, (f) 0.75, (g) 0.875, (h) 1, and (i) 1.125. When the lithium adsorption index corresponds to the values of 0.125 and 0.1875, five- and seven-link rings are formed in the silicene sheet. However, with an adsorption index of 0.25 or more, the Stone–Wales defects (combinations of five- and seven-link rings) are not formed. An increase in the number of adsorbed lithium atoms to an adsorption index of 0.75 and higher leads to the formation of a defect in the silicene sheet, caused first by a clearly visible lengthening of one of the Si-Si bonds, and then by the detachment of the Si atom from the silicene plate, which results in a five-membered silicon ring formation. A lithium atom enters the gap between the silicene sheet and the carbon substrate. Thus, a two-layer graphene substrate does not increase the strength of single-layer silicene with respect to lithium adsorption and even leads to a decrease in the specific capacity of the silicene anode. This is largely due to a low adhesion between silicene and bilayer graphene.

**Figure 4:**
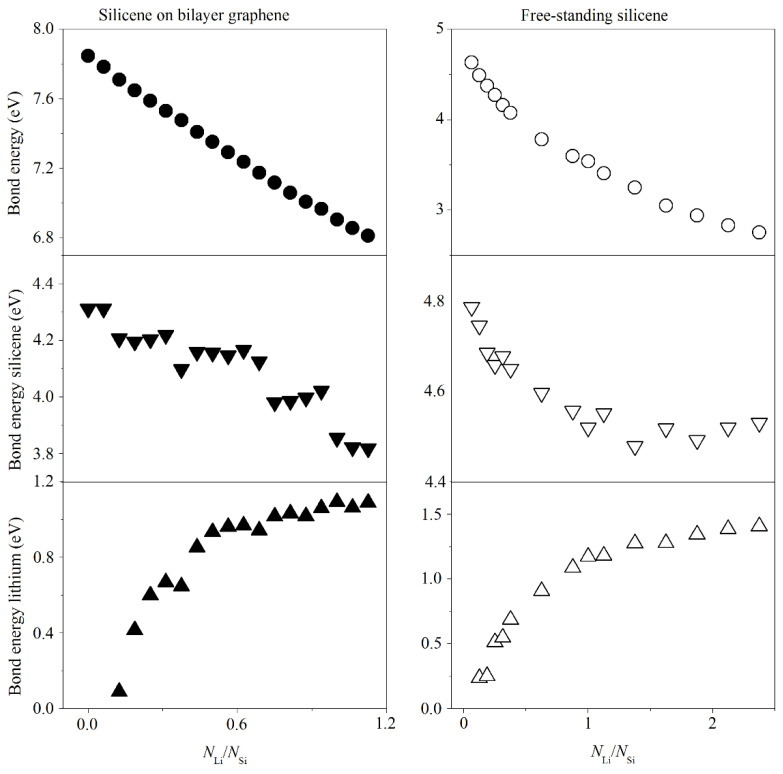
Bond energies between all atoms of the system, atoms in the silicene sheet and lithium coating, depending on the number of lithium atoms in the “silicene on bilayer graphene” system and for a free-standing silicene sheet.

Figure 4 shows the dependences of the bond energy between all atoms of the system, the bond energy between atoms in the silicene sheet, and the bond energy in the lithium coating on the number of adsorbed lithium atoms. It can be seen that as the number of lithium atoms in the system increases until an adsorption index reaches a value of 1.125, the total bond energy decreases by 13.2 and 26.4% for a silicene sheet on a graphite substrate and an autonomous silicene sheet, respectively. Basically, a decrease in the total bond energy occurs due to a decrease in the bond energy between silicon atoms in the silicene sheet. Thus, the bond energy in a silicene sheet upon adsorption of lithium with an adsorption index of 1.125 decreases by 11.5 and 4.9% for a silicene sheet on a carbon substrate and an autonomous silicene sheet, respectively. In the case of silicene on a carbon substrate, there is a stepwise decrease in the bond energy in the silicene sheet. The bond energies in the lithium coating are defined as 1.089 and 1.180 eV when the lithium adsorption index reaches 1.125 in the cases of silicene on a carbon substrate and free-standing silicene, respectively. A further increase in the number of adsorbed lithium atoms on free-standing silicene, when the index value reaches 2.375, leads to the increase in bond energy between lithium atoms in the coating by 19%. In the case of silicene located on bilayer graphene, the limiting value of the adsorption index is 1.125.

**Figure 5:**
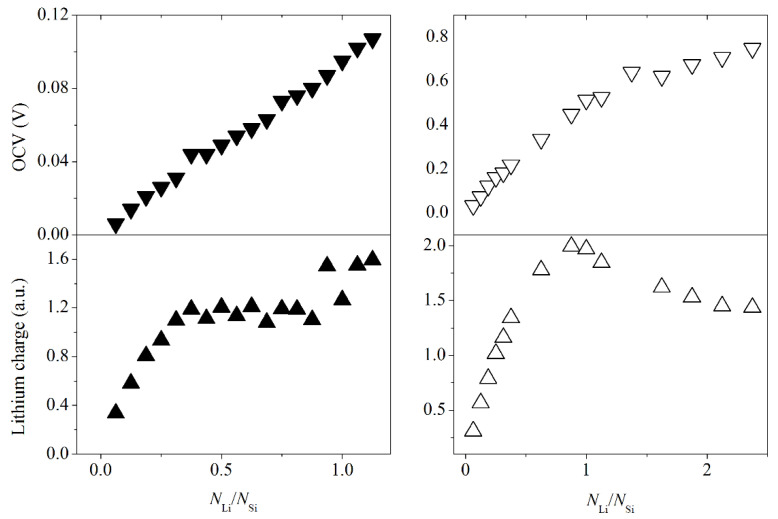
The open-circuit voltage and the total charge of lithium in the “silicene on bilayer graphene” system and for a free-standing silicene sheet.

Figure 5 shows dependences of the open-circuit voltage and the total Voronoi charge on lithium atoms in a silicene system on a carbon substrate and a free-standing sheet on the adsorption index. The open-circuit voltage in all considered cases does not exceed 1 V, i.e., the threshold, exceeding which can lead to the formation of metallic dendrites. The charge of the lithium subsystem in the silicene system on a carbon substrate increases from 0.336 to 1.187 a.u. as the adsorption index increases from 0.06 to 0.375. A further increase in the amount of adsorbed lithium to a ratio of 0.875 does not lead to an increase in the charge of the lithium subsystem. In systems with adsorption indices of 0.938, 1.06, and 1.125, the charge of the lithium coating increases to ~1.55 a.u. due to the ingress of lithium into the space between the silicene sheet and the carbon substrate. In autonomous silicene, the charge of the lithium coating reaches a maximum of 1.995 a.u. at the adsorption index of 0.875. Subsequently, the total charge of the coating decreases due to the lithium clustering.

Widely known characteristic features of graphene are a low, or even zero, density of electronic states at the Fermi level and a linear dependence of the energy on the wave vector in the vicinity of the k-point [25]. When two layers of graphene are combined, a small energy gap opens [26]. The existence of a tunable gap allows two-layer graphene to be used as low-power transistors. The intercalation of alkali metals into graphene leads to an increase in the carrier concentration and the density of states (DOS) at the Fermi energy level. Silicene is a semiconductor with a very narrow band gap (~37 meV) and a Dirac cone [27]. The alkali metals Li, Na, and K are primarily adsorbed on hollow areas of silicene without lattice distortion [28]. A significant charge transfer is observed from alkali metals to silicene, which causes silicene metallization. It can be assumed that when two semiconductors are fused, their band gap will take an intermediate value relative to the corresponding characteristics of the pure components. If this happened, then by changing the composition of the alloy, it would be possible to change the width of its band gap. This is not observed in the “silicene-on-bilayer graphene” system under consideration. Here, in the absence of alloy formation, the system as a whole acquires conductive properties, which is quite unusual.

**Figure 6:**
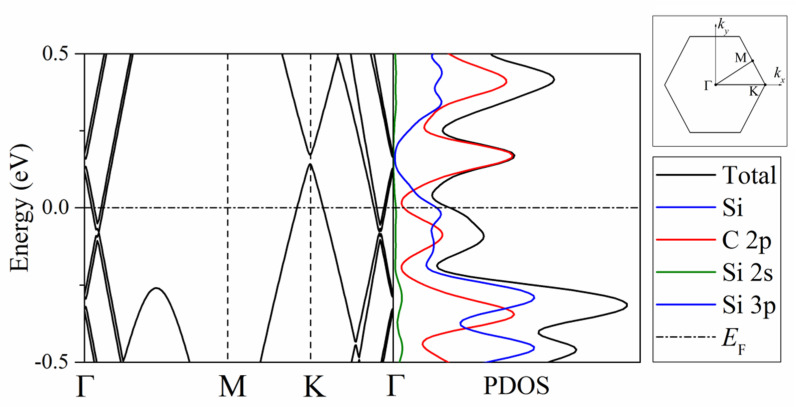
Band structure and partial spectra of electronic states of the “silicene on bilayer graphene” system; the Fermi level is indicated by a horizontal dashed line; the Brillouin zone for graphene with high symmetry points is shown in the inset at the top right.

Figure 6 shows the band structure and partial spectrum of electronic states of the system consisting of a silicene sheet on a carbon substrate. The top right insert shows the position of the high symmetry points in reciprocal space for graphene-like systems. The finite (nonzero) value of the density of electronic states at the Fermi energy level indicates that the system acquires the conductive properties. This system has conductive properties due to the interaction of 2p electrons of carbon with 3p electrons of silicon, which is consistent with the results obtained in [12]. In this case, the Dirac cone associated with the silicene subsystem in the band structure turns out to be raised above the Fermi level, while the graphene Dirac cone is located below the Fermi level. During the adsorption of lithium atoms, the silicene system on a carbon substrate does not change electronic properties and remains a conductor at all considered values of the adsorption index. At the same time, the adsorption of lithium on a free-standing silicene sheet, while the adsorption index reaches 1.125, leads to semiconductor–conductor transitions and vice versa. With a further increase in the amount of adsorbed lithium, the system remains a conductor. Similar transitions were observed during the adsorption of lithium in the silicene channel [8].

Two-dimensional van der Waals heterostructures, similar to the silicene–carbon structure studied here, are important for the creation of new optoelectronic devices [29]. Note that not only 2D, but also 3D structures of Si and C form a compound with well-defined sublattices for each element [30]. Both silicene- and silicene–graphene-layered composites are polar, two-dimensional (2D) materials. These materials have a high polarization density associated with their longitudinal optical (LO) phonon modes. As a consequence of this, long-range electric fields are generated in them. The emerging electron–phonon interaction has a significant effect on the structure, transport properties, and dispersion characteristics of optical phonons in these materials [31]. Long-range Coulomb interaction and electron shielding cause splitting between LO and transverse optical (TO) modes. The nature of this splitting depends on the dimension of the system. Analytical models of LO–TO splitting for 3D materials are still under development [32]. Similar studies should be done for 2D materials. 

We did not pursue the goal of investigating the properties of silicene on bilayer graphene required for its application in nanoelectronics. Therefore, their mutual rotations were not carried out in order to open the band gap in this system. However, the opening of the band gap in silicene is possible without its rotation. This can be done, for example, by placing silicene on a substrate made of a certain non-metallic material [33]. The fact is that the strong interaction of the metal with silicene spoils the electronic structure of this two-dimensional material. Various two-dimensional materials (h-BN, C-SiC, MoS_2_) have been investigated as a substrate for silicene intended for the needs of the electronics [34,35,36]. The van der Waals interaction between silicene and such a substrate breaks the symmetry of the silicene sublattices, which results in a band gap opening. The open band gap, as a rule, does not exceed 0.1 eV. Band gap adjustment is possible by rotating the silicene around the substrate, changing the distance from the silicene to the substrate, or by applying a perpendicular electric field.

## 4. Conclusions

In this work, we performed quantum mechanical calculations of the properties of a free-standing silicene sheet and a silicene sheet on a carbon substrate, which vary depending on the number of lithium atoms adsorbed onto them. We have investigated the structural, energetic and electronic properties of these systems. During the adsorption of lithium on the silicene sheet, the total bond energy and bond energies in the silicene sheet decrease in all cases under consideration. The calculated open-circuit voltages show that metal dendrites are not formed during the adsorption of lithium for all the considered adsorption indices. The addition of a carbon support increases the energetic stability of the system as a whole but decreases the stability of the silicene sheet. As a result, when using single-layer silicon on two-layer graphite as the LIB anode material, it is necessary to significantly limit the capacity of the anode. The adsorption of lithium onto a free-standing silicene sheet causes a semiconductor–conductor transition and a reverse transition. These transitions stop when the ratio of lithium to silicon reaches 1.125. With a further increase in the adsorption index, the system with a free-standing silicene sheet remains a conductor. At the same time, the system formed by silicene on a carbon substrate retains its conductive properties regardless of the number of adsorbed lithium atoms.

When the adsorption index reached 1.375, the breakdown of the autonomous silicene sheet was observed due to the incorporation of lithium between the silicon bonds. In the range of adsorption index values of ~0.12–0.19, five- and seven-membered rings are formed in a silicene sheet on a carbon substrate. In cases where the ratio of lithium to silicon is above ~0.87, a silicon atom detaches from the silicene sheet and a five-membered silicon ring is formed. Additionally, at values of the adsorption index above ~0.938, lithium has a possibility to enter the space between the silicene sheet and the carbon substrate. 

The destruction of the silicene sheet revealed during the simulation and the ingress of lithium into the gap between the silicene sheet and the carbon substrate are deficiencies that can create a problem for the application of this two-dimensional material as the anode of a lithium-ion battery.

## Figures and Tables

**Table 1:** Dependence of properties * of the “silicene on bilayer graphene” system on the number of adsorbed lithium atoms.

*N*_Li_/*N*_Si_	*E*^Li^_a_, eV	Si-Li, Å	Si-Si, Å	Li-Li, Å
0	-	-	2.282	-
0.0625	2.048	2.731	2.294	-
0.125	2.403	2.805	2.418	-
0.1875	2.022	2.843	2.380	-
0.25	1.743	2.743	2.355	-
0.3125	1.569	2.737	2.353	-
0.375	1.975	2.751	2.382	-
0.4375	1.406	2.772	2.381	-
0.5	1.291	2.751	2.379	-
0.5625	1.222	2.769	2.394	-
0.625	1.129	2.781	2.377	3.043
0.6875	1.142	2.736	2.481	2.799
0.75	1.204	2.781	2.427	2.845
0.8125	1.109	2.772	2.429	2.836
0.875	1.083	2.734	2.417	2.874
0.9375	1.067	2.802	2.395	2.973
1	1.098	2.768	2.415	2.897
1.0625	1.143	2.784	2.403	2.867
1.125	1.120	2.754	2.401	2.941

* *E*^Li^_a_ is the adhesion energy between lithium and silicene sheet on a carbon substrate; Si-Li denotes average bond lengths between silicon and lithium atoms; Si-Si denotes average bond lengths between silicon atoms; Li-Li denotes average distances between lithium atoms.

**Table 2:** Dependence of properties * of the “free-standing silicene sheet” system on the number of adsorbed lithium atoms.

*N*_Li_/*N*_Si_	*E*^Li^_a_, eV	Si-Li, Å	Si-Si, Å	Li-Li, Å	BG, eV
0.0625	2.154	2.762	2.313	-	M
0.125	2.219	2.800	2.326	-	M
0.1875	2.474	2.817	2.338	-	M
0.25	2.223	2.802	2.355	-	0.411
0.3125	1.961	2.770	2.362	-	M
0.375	1.858	2.737	2.376	-	0.474
0.625	1.574	2.721	2.417	3.056	0.134
0.875	1.417	2.721	2.459	2.956	0.409
1	1.390	2.726	2.475	2.968	0.645
1.125	1.212	2.735	2.465	2.979	M
1.375	1.227	2.723	2.481	2.957	M
1.625	0.871	2.773	2.487	2.961	M
1.875	0.772	2.757	2.478	2.970	M
2.125	0.652	2.774	2.486	2.979	M
2.375	0.601	2.769	2.476	2.926	M

* *E*^Li^_a_ is the adhesion energy between lithium and silicene sheet; Si-Li denotes average bond lengths between silicon and lithium atoms; Si-Si denotes average bond lengths between silicon atoms; Li-Li denotes average distances between lithium atoms; BG is the width of the band gap; M is metal.

## Data Availability

The data presented in this study are contained within the article.
